# Immunosurveillance encounters cancer metabolism

**DOI:** 10.1038/s44319-023-00038-w

**Published:** 2024-01-12

**Authors:** Yu-Ming Chuang, Sheue-Fen Tzeng, Ping-Chih Ho, Chin-Hsien Tsai

**Affiliations:** 1 Department of Fundamental Oncology, University of Lausanne, Lausanne, Switzerland; 2 Ludwig Institute for Cancer Research, University of Lausanne, Lausanne, Switzerland; 3 Graduate Institute of Life Sciences, National Defense Medical Center, Taipei, Taiwan; 4 Department and Graduate Institute of Biochemistry, National Defense Medical Center, Taipei, Taiwan

**Keywords:** Immunoediting, Immunometabolism, Cancer Evolution, Cancer, Immunology, Metabolism

## Abstract

Tumor cells reprogram nutrient acquisition and metabolic pathways to meet their energetic, biosynthetic, and redox demands. Similarly, metabolic processes in immune cells support host immunity against cancer and determine differentiation and fate of leukocytes. Thus, metabolic deregulation and imbalance in immune cells within the tumor microenvironment have been reported to drive immune evasion and to compromise therapeutic outcomes. Interestingly, emerging evidence indicates that anti-tumor immunity could modulate tumor heterogeneity, aggressiveness, and metabolic reprogramming, suggesting that immunosurveillance can instruct cancer progression in multiple dimensions. This review summarizes our current understanding of how metabolic crosstalk within tumors affects immunogenicity of tumor cells and promotes cancer progression. Furthermore, we explain how defects in the metabolic cascade can contribute to developing dysfunctional immune responses against cancers and discuss the contribution of immunosurveillance to these defects as a feedback mechanism. Finally, we highlight ongoing clinical trials and new therapeutic strategies targeting cellular metabolism in cancer.

## Abstract (web summary)

Metabolic deregulation and imbalance in immune cells within the tumor microenvironment drive immune evasion and compromise therapeutic outcomes This review highlights how metabolic crosstalk within tumors affects immunogenicity and promotes cancer progression.

## Graphical abstract

**Figure:**
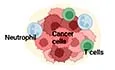

## Introduction

Understanding anti-tumor immunity and immune checkpoints has fostered the development of immunotherapies, such as immune checkpoint blockade (ICB) and, more recently, chimeric antigen receptor T-cells (CAR T-cells). However, the tumor microenvironment (TME) forms a metabolic barrier due to metabolic deregulations in tumor cells to disarm host anti-tumor immunity and current immunotherapeutic strategies (Ho and Liu, 2016; Li et al, 2019c). Metabolic dysregulation in tumors has been known for decades and is defined as a hallmark of cancer (Hanahan, 2022). In the past, those gained metabolic activities were considered to support unconstrained growth and anti-apoptosis in tumor cells.

In addition, metastatic tumor cells with unique metabolic abilities can potentiate their colonization at the secondary site (Altea-Manzano et al, 2020; Faubert et al, 2020; Tasdogan et al, 2020). Tumor cells have a high metabolic rate, but the tumor microenvironment often lacks proper blood flow and experiences changes in fluid pressure. Thus, it has been speculated that cells within the TME struggle to obtain nutrition and oxygen they need to survive (Heldin et al, 2004). However, several studies indicate that tumor cells can alter their metabolism to adapt to their environment and to support their bioenergetic needs (Bergers and Fendt, 2021). Additionally, tumor cells can change their physiological traits, such as metabolism and stemness properties, through interactions with immune and stromal cells to avoid detection by the immune system (Bayik and Lathia, 2021). Of note, tumor-derived metabolites can influence the makeup of metastatic niches and the behavior of T cells by rewiring the epigenetic landscape (Baumann et al, 2022; Chapman et al, 2020). Therefore, it can be inferred that tumor cells can alter the metabolic state of the TME or adapt their metabolism to overcome unfavorable conditions, such as nutrient scarcity and immune surveillance.

In this review, we discuss the effects of bidirectional metabolic crosstalk between tumor cells and infiltrating immune cells on immune suppression and metabolic adaption of cancer cells during immunosurveillance derived from the innate and adaptive arm of the immune system, with a particular focus on T cells, regulatory T cells (Tregs), and natural killer (NK) cells.

## Metabolic reprogramming shapes tumorigenesis

### Intrinsic mutations drive specific nutrient dependencies during cancer progression

**Figure 1:**
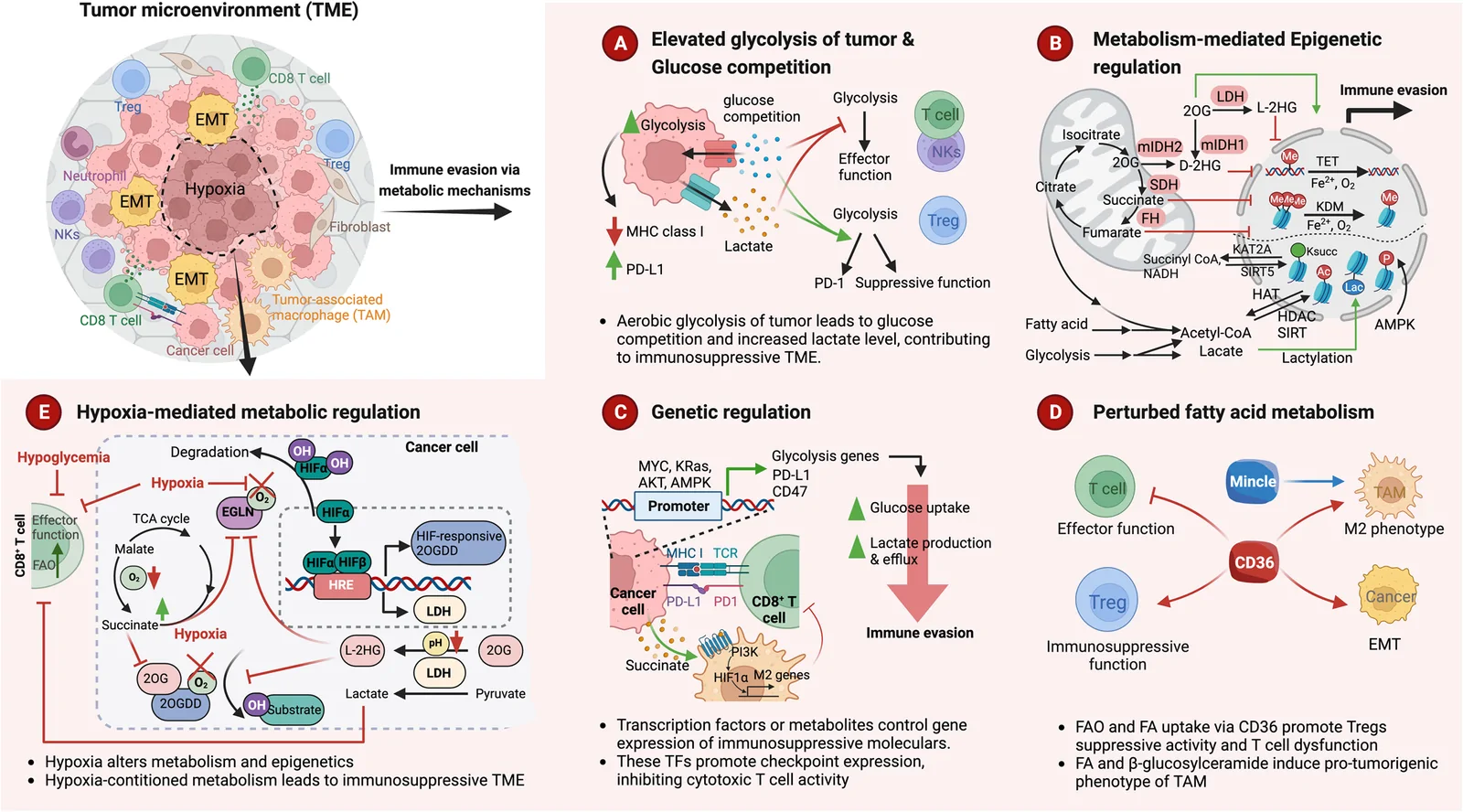
Mechanisms of metabolic tumor immune evasion. During cancer progression, tumor cells and tumor-infiltrating lymphocytes (TILs), including CD8^**+**^ T cells, regulatory T cells (Tregs), and natural killer (NK) cells, rewire their metabolic programs in response to microenvironmental stress, such as nutrient deprivation and hypoxia. The metabolic interaction between tumor cells and diverse immune cells in and around solid tumors orchestrates the immunosuppressive TME and restrains host anti-tumor immunity. As a feedforward response to a stressful microenvironment, cancer cells acquired different metabolic adaption and interaction mechanisms to evade immune surveillance. It is also likely that harnessing the adaptive and innate immune response might bring greater rewards in cancer therapy. (**A**) Metabolic transitions can cause effector lymphocytes to become exhausted and dysfunctional, which in turn affects their differentiation. NK and T cells rely on glycolysis to maintain their effector function and viability. Proliferating tumor cells with elevated glycolysis may affect MHC class I and PD-L1 protein expression levels and compete with surrounding immune cells for glucose. This competition leads to glucose deprivation and elevated lactate levels, which impair the effector function of NK and CD8^**+**^ T cells but become beneficial for the suppressive activity of Tregs. (**B**) Cells metabolize nutrients such as glucose, amino acids, and fatty acids to produce various metabolites, like ATP, acetyl-CoA, NAD^**+**^, SAM, α-KG, fumarate, and succinate. These metabolites function as substrates or cofactors for modifying proteins and chromatin. Histone acetyltransferases (HATs) catalyze histone acetylation, while lysine deacetylases (HDAC and SIRT) mediate the reverse reaction. Glycolysis, fatty acid metabolism, and TCA cycle contribute to acetylation modification. The production of lactate generates lactyl-CoA, which contributes a lactyl group to lysine residues of histone proteins, creating a novel modification called lactylation. Succinyl-CoA, the primary substrate for succinylation, is derived from the TCA cycle, and KAT2A, CPT1A, and SIRT5 mediate the opposite reaction. AMPK is required for histone phosphorylation, depending on the ATP: AMP ratio. Chromatin methylation is linked to the folate cycle and the methionine cycle. Succinate, fumarate, and 2-HG inhibit KDMs and TETs, which catalyze demethylation in an α-KG-dependent manner. Additionally, NAD^+^ and NADH transitions lead to epigenetic modifications such as acetylation and succinylation. (**C**) Certain transcription factors, as well as oncogenic signaling pathways such as MYC, KRAS, AKT, and AMPK, are responsible for regulating the expression of immune checkpoint molecules like CD47 and PD-L1 as well as glycolysis-related genes, which, in turn, lead to immune evasion. Additionally, metabolites can also directly impact the expression of immunosuppressive molecules. For instance, succinate can activate PI3K-HIF1 signaling and promote M2 polarization by binding to succinate receptors. (**D**) Distinct metabolic preferences have been observed among tumor-infiltrating lymphocytes (TILs), which greatly impact their function and overall status. The maintenance of suppressive function in Tregs and M2 macrophages relies heavily on oxidative phosphorylation (OXPHOS) and fatty acid (FA) oxidation, facilitated by fatty acid transporters like CD36. The pro-tumorigenic phenotype of macrophages is also attributed to mincle-dependent β-glucosylceramide efflux. In contrast, the presence of fatty acids in the tumor microenvironment impairs the effector function and viability of intratumoral CD8 T cells. (**E**) The extent to which a 2-oxoglutarate-dependent dioxygenase (2OGDD) is suppressed by hypoxia depends on several factors, such as its expression level, oxygen affinity, and sensitivity to succinate and L-2HG inhibition. Hypoxia decreases the activity of hypoxia-sensitive 2OGDD, including EGLN proline hydroxylases. This inhibition of EGLN leads to the activation of HIF transcriptional activity. Moreover, hypoxia upregulates HIF target genes, including lactate dehydrogenase (LDH) and 2OGDD subsets. LDH generates L-2HG, which is potentiated by hypoxia and cellular acidosis, thereby accumulating high level of L-2HG. In certain types of cells, severe hypoxia may dysregulate the tricarboxylic acid (TCA) cycle, resulting in increased succinate production. Both succinate and L-2HG, which build up under hypoxic conditions, inhibit the function of 2OGDD. KSUCC Succinylated Lysine, FA fatty acid, FAO fatty acid oxidation, TAM tumor-associated macrophage, 2OG 2-oxoglutarate, HRE HIF-responsive element.

Several oncogenic mutations that dictate nutrient requirements of cancer cells among different cancer types and organs have been identified (Fernandez-Garcia et al, 2020; Nagarajan et al, 2016). Alterations in oncogenes and tumor suppressor genes regulate amino acid homeostasis and cellular response to nutrient stress, contributing to an immune profile shift within a tumor and to the response to immune therapy (Gwinn et al, 2018; Kao et al, 2022). For example, activating the PI3K-AKT signaling pathway in various cancers enables cells to enhance nutrient uptake and to increase the biosynthesis of biological macromolecules, even in cases of low extracellular growth factors (Goncalves et al, 2018). Tumors with mutant K-RAS can resist nutrient deprivation during hypoxia in vivo (Garcia-Bermudez et al, 2022) and also can evade immune surveillance by modulating CD47 and neutrophils-recruiting chemokines, e.g., CXCL8 (Hu et al, 2023; Sparmann and Bar-Sagi, 2004). Additionally, oncogenic activation of MYC, AKT, and K-RAS can contribute to PD-L1 expression of tumor cells, which inhibits immune surveillance (Casey et al, 2016; Coelho et al, 2017; Parsa et al, 2007). In addition to oncogenic mutations, p53, a common tumor suppressor gene, is often mutated or deleted in approximately 50% of human cancers, which leads to aberrant metabolic reprogramming in cancer cells (Kruiswijk et al, 2015). Loss of function of PTEN in cancer increases autophosphorylation of phosphoglycerate kinase 1 (PGK1), enhancing glycolytic activity and promoting brain tumor formation (Qian et al, 2019). As a result, intrinsic oncogenic dysfunction accompanies robust changes in nutrient requirements and the immune landscape of the TME (Fig. 1).

Furthermore, mutations in metabolic enzymes can directly impact metabolic phenotypes and cancer aggressiveness through epigenetic regulation (Fig. 1). For instance, IDH1 and IDH2 mutations are frequently found in gliomas and can produce D-2 hydroxyglutarate (D-2HG). This, in turn, can cause epigenetic changes by affecting the DNA demethylation status of 5mC (5-methylcytosine) and its variants. In clinical samples from human patients with IDH1 mutant gliomas, it was found that CD8^**+**^ T cells take up tumor-derived D-2HG, which changes their metabolic program. This decreases antitumor functions by impairing cytotoxicity and interferon-γ signaling (Notarangelo et al, 2022). Mutations in the succinate dehydrogenase (SDH) and fumarate hydratase (FH) genes lead to the accumulation of succinate and fumarate, respectively, and also control the 2-oxoglutarate-dependent oxygenase superfamily, including histone lysine demethylase enzymes (KDMs), TET methylcytosine dioxygenases, and hypoxia-inducible factor (HIF) hydroxylases by competing with α-ketoglutarate (Xiao et al, 2012). Therefore, cancer cells engage metabolic flexibility through genetic mutations or epigenetic modification to facilitate tumor aggressiveness.

### Tissue origin determines metabolic preference

The in situ environment of each tissue also reshapes the metabolic preferences of tumor cells even carrying the same mutations. Pan-cancer transcriptomic analyses show that the metabolic profile of the primary tumor is very similar to that of the corresponding normal tissue (Gaude and Frezza, 2016). Tumors caused by the same oncogene but originating from different tissues can exhibit varying metabolic patterns. For instance, hepatocarcinoma (HCC) with MYC amplification diminishes glutamine anabolism, while MYC-induced lung adenocarcinoma engages glucose catabolism and increases glutamine levels (Yuneva et al, 2012). It has been observed that similar genetic defects such as K-RAS activation and p53 loss in mouse non-small cell lung cancer (NSCLC) and pancreatic cancer cause a bifurcation of amino acid metabolism, indicating that metabolic reprogramming is dependent on the in-situ tissue (Mayers et al, 2016; Sullivan et al, 2019). Interestingly, tumor cells interacting with surrounding stroma cells can sculpt metabolic preferences to facilitate metastasis. For instance, adipocytes fuel fatty acids for ovarian cancer to boost metastatic tumor cell growth at distant sites via elevated fatty acid-binding protein 4 (FABP4) (Nieman et al, 2011). Compared to other tissues, the lactate expression level in the lung tissue is elevated, even under well-oxygenated conditions (Fisher, 1984). This means lactate is readily available in the lung environment and can be used as a carbon source to help NSCLC growth (Faubert et al, 2017). These findings indicate that even though tumor cells retain some metabolic characteristics of their tissue of origin, different microenvironmental features can drive various metabolic adaptations (Gaude and Frezza, 2016). Thus, oncogenic mutations drive nutritional requirements during cancer progression, while nutrient availability in local organs is a critical factor contributing to metabolic preference and metastatic growth.

On the other hand, different intrinsic oncogenic signaling could elicit various metabolic changes even in the same tissue type. For example, MYC amplification-driven HCC relies on aerobic glycolysis and glutaminolysis, whereas HGF/cMet-driven HCC utilizes glucose for glutamine anabolism (Yuneva et al, 2012). Overall, oncogenic mutants can cooperate with local surroundings to redefine oncogene-imposed metabolic dependencies of cancer cells and influence tumor development.

### Metabolic plasticity and flexibility of cancer

Metabolic plasticity in tumor cells promotes their survival in response to stress. Tumor cells can survive nutrient deserts by activating a replenished metabolic route. It has been observed that the metabolic flexibility of tumor cells is beneficial in liver and lung cancer, which alternatively engage fatty acid desaturation to support survival (Vriens et al, 2019). In the case of glutamine deprivation, tumor cells can use aspartate metabolism by inducing aspartate/glutamate transporter SLC1A3 expression, which helps them to maintain the electron transport chain and to continue to synthesize glutamate, glutamine, and nucleotides (Tajan et al, 2018). Additionally, hepatocytes are responsible for synthesizing ketone bodies, which normal adult hepatocytes cannot consume. When there are no nutrients available, HCC cells can increase the expression of OXCT1 (3-oxoacid CoA transferase 1), which enables them to break down ketone bodies and generate energy (Huang et al, 2016).

Tumor cells have a unique capacity for waste recycling that differs from normal metabolic processes (Li et al, 2019a). The urea cycle is mainly used to eliminate ammonia, a metabolic waste, from the body. However, deregulation of the urea cycle occurs in many tumors by increasing the expression of related enzymes to enhance pyrimidine synthesis. This accompanying imbalance of the pyrimidine-purine ratio results in transversion mutations in the genome (Lee et al, 2018). Moreover, intermediates from the urea cycle can be transferred to other biosynthesis processes (Keshet et al, 2018). For example, ammonia can serve as a nitrogen source for proline and aspartate synthesis to fuel breast cancer progression (Spinelli et al, 2017). Typically, ammonia-derived carbamoyl phosphate (CP) cannot be used for polyamine biosynthesis and nucleotide synthesis. However, upregulation of the carbamoyl phosphate synthase 1 (CPS1) has been found to increase CP production in NSCLC, HCC, and colon cancer. The excess of CP can subsequently enter the pyrimidine synthesis pathway to sustain tumor cells with rapid proliferation (Kim et al, 2017; Li et al, 2019a). These findings reveal a link between mutation, cancer context, and metabolic flexibility in controlling tumor progression.

### Metabolic properties of metastases and the metastatic niche

**Figure 2:**
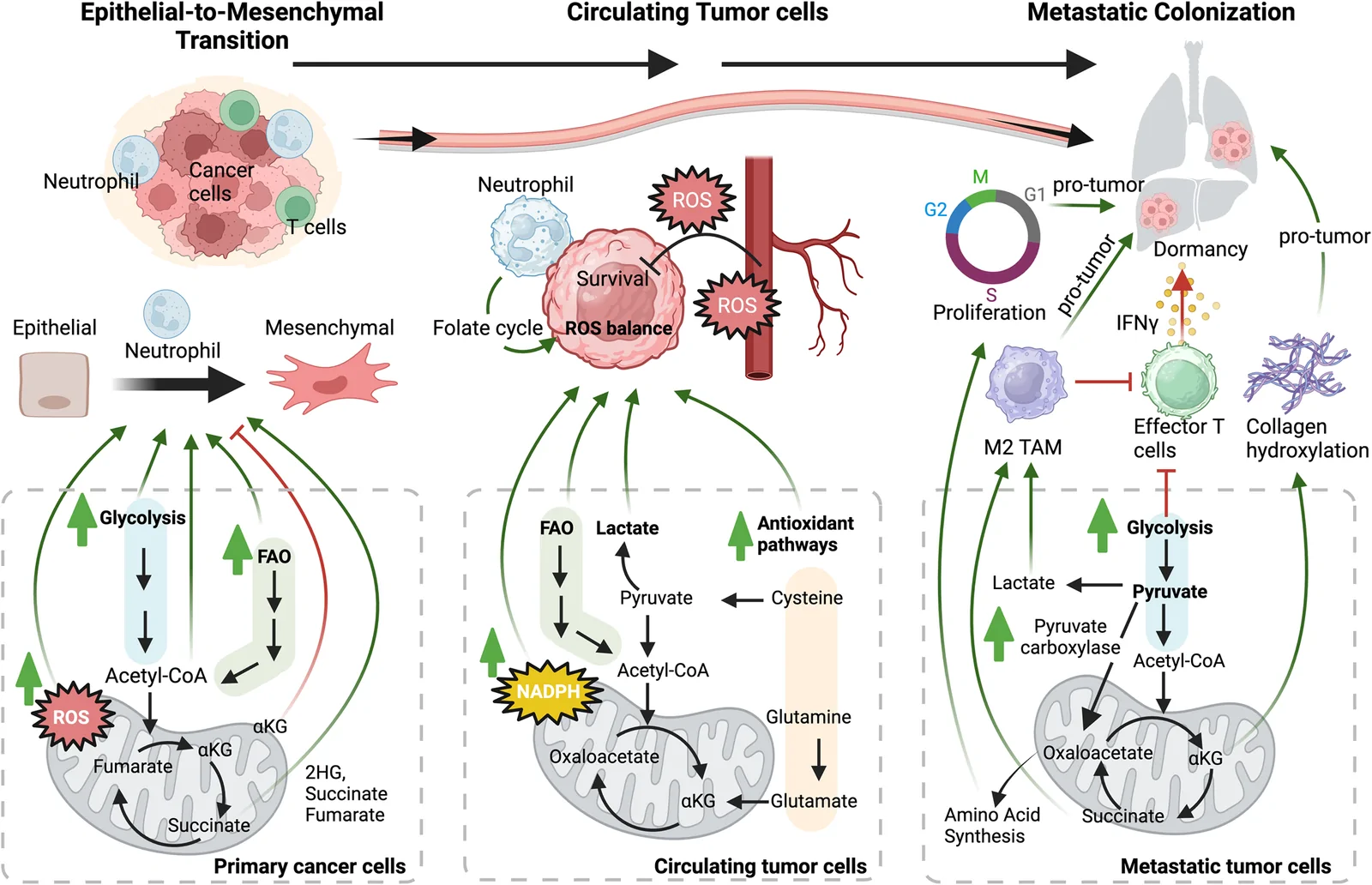
Metabolic plasticity in metastasis. Cells undergo EMT to increase mobility, travel in circulation, and develop metastases, which can be regulated by metabolic activity. Metastasis involves rearranging the cytoskeleton and releasing enzymes that promote glycolysis for proliferation. Certain metabolites, such as fumarate, succinate, and fatty acid, act as signaling molecules that support EMT (left panel). Once detached, cancer cells also produce mitochondrial ROS, which promotes EMT and metastatic potential. When cancer cells are circulating (center panel), they may die due to oxidative stress, but those that survive have a metabolic advantage, which can be promoted in combination of neutrophils. CTCs increase NADPH production and lactate uptake, which boosts their antioxidant capacity. Once cancer cells reach the metastatic site (right panel), they must alter the tumor microenvironment to suppress immune surveillance, which can be done metabolically by facilitating TAM polarization and effector T cell dysfunction. Additionally, cytokines and metabolites likely influence metastatic niche development (e.g., collagen hydroxylation), cancer cell dormancy, and cell proliferation. Thus, the metabolic cascade plays a critical role in this process and may offer a therapeutic vulnerability for treating metastatic cancer.

Aggressive tumor cells usually undergo epithelial-mesenchymal transition (EMT) to spread and create metastases, and EMT is associated with significant metabolic rewiring. Recent studies have identified specific metabolic pathways contributing to EMT, such as increased glycolysis and TCA activity (Jia et al, 2021; Fig. 2). Increased glycolysis in tumor cells is known to support cytoskeleton rearrangements during the EMT process (Parlani et al, 2023). Certain cofactors for epigenetic modifiers, such as succinate and fumarate addressed above, can also trigger EMT (Colvin et al, 2016; Letouze et al, 2013; Sciacovelli et al, 2016; Wu et al, 2020). Acetyl-CoA, another cofactor for post-translational modifications and an epigenetic modifier, has also been reported to be involved in EMT (Lu et al, 2019; Qin et al, 2019). On the other hand, increasing alpha-ketoglutarate (α-KG) levels can boost TET activity and prevent EMT by demethylating miR-200 family promoter regions (Atlante et al, 2018). Beta-oxidation also participates in epigenetic regulation of EMT-related genes through acetylation of histones using acetyl-CoA, ultimately determining cell metastasis (Loo et al, 2021). In addition, an increase of the fatty acid transporter CD36 results in lipid uptake, which in turn promotes EMT (Nath et al, 2015). Similarly, AMPK activation is often found in metastatic tumor cells, which preserve NADPH pools by skewing lipogenesis versus beta-oxidation. (Jeon et al, 2012).

Once tumor cells leave the primary site and embark on the metastatic journey, these circulating tumor cells (CTCs) must adapt their metabolism to cope with various stresses (Ubellacker et al, 2020). While detachment of cancer cells causes a spike in reactive oxygen species (ROS), CTCs must counteract ROS, suggesting that metabolic reprogramming is required to support metastasis (Schafer et al, 2009). It was observed that successfully metastasizing melanomas undergo reversible metabolic alterations to help tumor cells to survive in the circulation or visceral organs. One such alteration is using the folate pathway to generate NADPH, which increases their ability to withstand oxidative stress compared to tumor cells in primary subcutaneous sites (Piskounova et al, 2015). Furthermore, CTCs can pass through lymphatic system to acquire the capacity for overcoming ferroptosis, a lipid ROS-dependent cell death, and enhance their survival (Ubellacker et al, 2020). Similarly, the dependence of gastric cancer cells on fatty acid oxidation has been coupled to increased antioxidant defense by increasing NADPH generation (He et al, 2019). In addition, CTCs can form clusters with neutrophils in circulation to enhance survival and proliferation by upregulating gene signatures associated with cell cycle, DNA replication, pyrimidine metabolism, and folate biosynthesis (Szczerba et al, 2019).

A raised glutamine metabolism also quenches ROS to compromise oxidative stress by replenishing the mitochondrial NADPH pool through glutathione generation (Gong et al, 2022). Indeed, glutamine can facilitate a stem cell phenotype by maintaining the redox status (Liao et al, 2017). Consistently, dietary antioxidants can decrease ROS and promote metastasis (Le Gal et al, 2015; Sayin et al, 2014). Additionally, RAS activation can redirect glutamine metabolism to balance cellular ROS by elevating the levels of cystine/glutamate antiporters (Lim et al, 2019). Thus, antioxidant metabolic precursors such as cysteine and glutamine maintain redox balance in CTCs and combat oxidative damage to promote survival (Combs and DeNicola, 2019). These studies suggest that enhanced antioxidant pathways after dissemination provide additional benefits, while targeting ROS balance by manipulating cellular metabolism may improve the current treatment in reducing tumor metastasis.

Since nutrient abundance and oxygen level are known to vary in different organs, metastatic tumor cells increase metabolic plasticity by using alternative nutrients and second metabolites to fuel energy metabolism and biosynthetic pathways. Increasing evidence indicates that primary tumors and their metastatic counterparts exhibit distinct metabolic features, suggesting that the local environment can affect metastatic fitness. A metastatic phenotype of breast cancer drives bioenergetic flexibility by enhancing glycolytic activity and mitochondrial functions in cancer cells in a peroxisome proliferator-activated receptor-gamma coactivator 1α (PGC1α)-dependent manner (Andrzejewski et al, 2017). Similarly, CTCs of prostate cancer characterized by increased glycolysis metabolic enzymes, such as HK2, PDK1, and PKM2, exhibit strong metastatic capacity (Chen et al, 2018). In contrast, metastatic liver cells show a preferences for aerobic glycolysis and a reduction of mitochondrial dependency, which is caused by the dysregulation of pyruvate dehydrogenase kinase-1 (PDK1) (Dupuy et al, 2015). Metastatic melanoma cells elevate NADPH and consume lactate to resettle glucose usage, redirecting glucose carbon into the oxidated PPP and enhancing their antioxidative capacity (Tasdogan et al, 2020). Furthermore, stage-dependent differential requirements for anaplerosis have been documented, showing increased pyruvate carboxylase (PC) in tumors of NSCLC patients compared to adjacent normal tissue (Sellers et al, 2015). Intriguingly, lung metastases of breast cancer elevate PC for supporting TCA cycle compared to primary tumors (Christen et al, 2016). It was found that lung metastases displayed higher pyruvate levels, the substrate of pyruvate carboxylase (PC), due to increased mTORC1 signaling. As a result, lung metastases are more sensitive to mTORC1 inhibitors than the original tumors (Rinaldi et al, 2021). Accordingly, genetic abolishment of PC suppresses pulmonary metastasis but does not affect metastatic growth in non-pulmonary organs (Shinde et al, 2018). Those studies highlight that pyruvate metabolism and PC activity may be the cues of organ tropism.

Metabolites also contribute to the development of metastatic niches (Fig. 2). For instance, cancer cells can secrete succinate into the extracellular milieu to induce tumor associated macrophage (TAM) polarization, which promotes EMT (Wu et al, 2020). An in vivo metabolic analysis of the secretome in lung cancer models revealed increased succinate in the plasma, which induces macrophage-dependent cytokine expression and downstream signaling in the metastatic niche, stimulating cancer cell migration and metastasis (Wu et al, 2020). In breast cancer, pyruvate is also involved in remodeling the metastatic niche in lung tissues by stimulating the production of α-KG, which serves as a metabolic activator of collagen prolyl-4-hydroxylase and leads to collagen hydroxylation. Similarly, glutamine dehydrogenase (GDH) increases in metastatic lesions, which converts glutamate into α-KG, particularly under hypoxia and hypoglycemia. Patients with high GDH expression have poor overall survival (Liu et al, 2015). Moreover, inhibiting pyruvate uptake or targeting alanine aminotransferase 2 activity to prevent pyruvate-to-α-KG conversion decreases collagen hydroxylation and metastatic growth (Elia et al, 2019). This suggests that metabolite availability appears to be critical for driving metastasis, highlighting the potential of therapeutic interventions by targeting metabolic vulnerabilities.

## Cancer cell–TIL interactions contribute to metabolic reprogramming and immune evasion

### Glucose competition in tumors drives an immunosuppressive TME

With a more detailed understanding of the crosstalk of nutrients and metabolites between tumor and non-tumoral cells in the TME, dysregulated energetics would not be stereotypically considered the alternative way to fuel energy usage for tumor cells (Fig. 1). As cancer progresses, there may be a shortage of glucose and oxygen due to competition between immune cells and tumor cells. Studies have shown that glycolytic transcriptional programs in tumor cells are linked to an immunosuppressive TME by recruiting suppressive myeloid cells (Li et al, 2018). Oxygen- and glucose deprivation decrease MHC class I antigens in cancer cells, causing them to become unresponsive to IFN-mediated cytotoxic effects due to a malfunctioning STAT1 (Marijt et al, 2019). Notably, the gain of metabolic reprogramming in transformed cells also affects the magnitude of the immune response (Li et al, 2019c). Moreover, if there is a lack of glucose, the activity of tumor-infiltrating T lymphocytes (TILs) is metabolically compromised and functionally exhausted in the TME (Chang et al, 2015; Ho et al, 2015). In contrast, multiple studies suggest that increased glycolysis may hinder the induction of Treg cells, whereas impeded glycolysis promotes the expression of Foxp3 upon IL-2 and TGF-β stimulation (Michalek et al, 2011; Shi et al, 2011). NK cells also rely on glycolysis to sustain their effector function and viability (Cong et al, 2018). Mechanistically, NK cells gradually elicit their dysfunction by inhibiting glycolysis and impairing viability via TGFβ-mediated fructose-1,6-bisphosphatase (FBP1) expression (Cong et al, 2018). Additionally, a similar study indicates that the dysfunction of NK cells in the TME is caused by the suppression of glucose metabolism due to lipid peroxidation-associated oxidative stress (Poznanski et al, 2021). These findings indicate that glucose availability or glycolysis activity in the TME is crucial for maintaining the effector function of T cells and NK cells.

Lactate dehydrogenase A (LDHA) is known to be highly expressed in glycolytic tumor cells, where it converts excess pyruvate and NADH into lactate and NAD^+^ to sustain the ATP-generating arm of glycolysis. Indeed, serum LDHA levels are a clinical prognosis markers that reflect poor survival and aggressiveness (Brand et al, 2016; Gallo et al, 2015). Based on current evidence, lactic acid, a by-product of glycolysis, has been found to have an immune suppressive function that may cause the failure of immune checkpoint blockade (Certo et al, 2021). It is now well-appreciated that lactate accumulation in highly glycolytic tumors suppresses the cytotoxic function of TIL, leading to immune escape (Brand et al, 2016). Lactic acid in the TME partially facilitates the downregulation of genes related to NAD^+^ salvage metabolism, impairing cytotoxicity, and viability of NK cells (Guo et al, 2022). In contrast, highly glycolytic tumors can cultivate a metastatic niche with high lactate levels to suppress T-cell activation and NK cell function (Huber et al, 2017; Payen et al, 2020). High lactate expression also stabilizes HIF1α in macrophages to promote the expression of a HIF1α-stabilizing lncRNA, which is then transported back to tumor cells via extracellular vesicles. This forms a feedforward loop that fuels tumor growth and suppresses T-cell/NK function (Chen et al, 2019). Tregs utilize lactate to sustain their suppressive function; when lactate utilization is impaired, it results in the loss of suppressive function and proliferation (Watson et al, 2021). In highly glycolytic tumors, such as MYC-amplified tumors and liver tumors, Treg cells actively enhance the expression of PD-1 to compromise the response of ICB through monocarboxylate transporter 1 (MCT1)-mediated uptake of lactic acid, whereas the expression of PD-1 by effector T cells is dampened in a lactate-enriched TME (Kumagai et al, 2022). In addition, lactate differentially impacts tumor-associated macrophages (TAMs) by harming the metabolism of anti-tumoral TAMs but supporting the suppressive functionality of pro-tumoral TAMs as well as their metabolic activity (Geeraerts et al, 2021). Importantly, recent studies have shown that histone lactylation reveals a new way to link nutrient metabolism to gene regulation (Zhang et al, 2019) and gene function as a metabolic-epigenetic alternative hub in macrophage polarization (Noe et al, 2021).

In contrast to the deprivation of glucose from activating T cells, acidic tumor microenvironments do not favor lactate export from T cells via MCT1, which halts T-cell expansion. An inverse relationship between glucose metabolism and T-cell infiltration has been described in cancer (Ottensmeier et al, 2016). Acidosis causes pro-tumorigenic neutrophils to become active, which augment acidity by exporting protons following their oxidative burst (Martinez et al, 2006). The metabolic symbiosis between lactate-producing and lactate-consuming cells within tumors is linked to acquired resistance against anti-angiogenic therapy and immune system responses to tumors (Allen et al, 2016; Jimenez-Valerio et al, 2016; Pisarsky et al, 2016).

### Amino acid metabolism and immunosurveillance

Except for glucose, the availability of amino acids can also impact lymphocyte differentiation and function. Within the tumor, glutamine can be consumed by effector T cells and glutamine-addicted cancer cells, indicating a metabolic competition for glutamine in the TME (Carr et al, 2010; Yang et al, 2021). Glutamine uptake, which fuels mTORC1 activity, is essential for differentiating T cells into inflammatory effector T cells (Nakaya et al, 2014; Sinclair et al, 2013). Deprivation of extracellular glutamine steers differentiation of naïve CD4^**+**^ T cells into Tregs even in conditions favoring the generation of other T cell subsets, whereas supplementation with the glutamine-derived metabolite α-KG supports differentiation towards Th1 through mTORC1 regulation (Klysz et al, 2015). In contrast, selective depletion of glutamine metabolism in cancer cells improves proliferation and cytokine production of effector T cells in triple-negative breast cancer models (Edwards et al, 2021). In addition, glutamine withdrawal, but not glutaminolysis inhibition, in NKs results in impaired cell growth and effector function due to loss of MYC (Loftus et al, 2018). Previous studies showed that increased arginine availability could sustain T-cell anti-tumor immunity and synergistic effects of checkpoint blockade therapy (Geiger et al, 2016). The depletion of arginine and tryptophan in cell cultures stimulates Treg generation (Cobbold et al, 2009). Activation of T cells leads to increased L-arginine metabolism mediated by arginase 2, which contributes to the generation of central memory-like T cells endowed with higher survival capacity and anti-tumor immunity. This suggests that additional arginine-sensing pathways also contribute to T cell survival (Geiger et al, 2016). On the other hand, cancer cells can sustain their survival in arginine starvation conditions by inducing ATF4-dependent upregulation of argininosuccinate synthetase 1 (ASS1), allowing de novo arginine synthesis to disrupt T cell function and chromatin remodeling (Crump et al, 2021). Consequently, the accumulation of extracellular lactate and the deprivation of certain amino acids in the tumor environment could affect immune cell proliferation, function, and differentiation.

Distinct metabolic preferences have been shown in tumor-suppressing macrophages (M1) and tumor-promoting macrophages (M2); for example, M1 macrophages upregulate glycolysis, whereas M2 macrophages upregulate OXPHOS and fatty acid oxidation (FAO) (Mills and O’Neill, 2016). The metabolic interplay in the TME has also been described for macrophages and myeloid-derived suppressor cells (MDSCs) (Vitale et al, 2019). Hypoxic impaired glycolysis activity in TAMs has been shown to cause endothelial dysfunction, consequently leading to metastases and nutrient availability. (Wenes et al, 2016). Tumors can also upregulate key catabolic enzymes like ARG1 or indoleamine 2,3-dioxygenase 1 (IDO1) in myeloid cells to deplete arginine and tryptophan within a tumor, which are crucial for regulating T-cell differentiation and proliferation (Mondanelli et al, 2019). Tumor-derived lactate polarizes macrophages towards a pro-tumorigenic M2 cell fate that induces arginase-1 to deprive arginine in T and NK cells (Colegio et al, 2014; Vitale et al, 2019). Arginine could be used in M1 and M2 polarized macrophages but in different metabolic processes. M1 and M2 macrophages use inducible nitric oxide synthase (iNOS) and arginase 1 (Arg1), respectively, for arginine catabolism (Modolell et al, 1995). The metabolite, nitric oxide (NO) from iNOS, could contribute to anti-tumor activity, but the metabolites from Arg1 rather promote tumor cell growth and inhibit NO production (Chang et al, 1998, 2001). Additionally, glutamine usage also participates in regulating M2 macrophages, showing that inhibition of the glutamine synthetase can polarize M2 macrophages towards an M1-like phenotype (Palmieri et al, 2017). These data suggested that immune cell expansion and differentiation depend on environmental nutrient availability, which controls an immunosuppressive tumor microenvironment.

### Fatty acid and lipid metabolism in immune escape

In addition to glucose and amino acids, tumor cells also perturb fatty acid (FA) levels and types in the TME (Pavlova and Thompson, 2016; Fig. 1). Tumor cells engage in de novo synthesis of fatty acids, which is mainly contributed by ATP-citrate lyase (ACLY), acetyl-CoA carboxylase (ACC), and fatty acid synthase (also known as FASN) (Mashima et al, 2009). With the enrichment of lipid droplets and extracellular FAs in the tumor microenvironment, recent studies indicate that tumor-infiltrating leukocytes could undergo metabolic reprogramming by using lipid compartments to adapt to metabolic constraints. Metabolic stress causes CD8^**+**^ TIL exhaustion, but FA catabolism can sustain their effector function in hypoglycemia and hypoxia conditions through peroxisome proliferator-activated receptor (PPAR)-α signaling (Zhang et al, 2017). Linoleic acid also helps to improve the metabolic fitness of CD8^**+**^ T cells, which directs them away from exhaustion and towards a memory-like phenotype (Nava Lauson et al, 2023). But, the utilization of oxidized lipids may trigger the accumulation of lipid-related ROS, which dampens CD8 T cell effector function by increasing levels of the scavenger receptor CD36 (Ma et al, 2021; Xu et al, 2021). Tregs are reported to preferentially use FAO (Michalek et al, 2011) or microbe short-chain fatty acids to control Treg differentiation (Smith et al, 2013). Different from CD8 T cells, Tregs increase the usage of FA through CD36, which sustains their survival and immunosuppressive function (Wang et al, 2020). Mechanistically, CD36 is selectively upregulated in intratumoral Treg cells and enhances mitochondrial fitness via peroxisome proliferator-activated receptor-β (PPARβ) signaling, rewiring Tregs to adapt to a lactate-enriched tumor microenvironment (Wang et al, 2020). Higher percentages of Treg cells expressing CD36 and SLC27A1 are observed in brain tumors. Inhibiting fatty acid transport with sulfo-N-succinimidyl oleate (SSO) or FAO with etomoxir impairs the immunosuppressive capabilities of Tregs (Miska et al, 2019). Tregs rely on lipogenesis to accumulate intracellular lipids and complement glycolysis for their function and expansion (Pacella et al, 2018; Zeng et al, 2013). In addition, the availability and usage of fatty acids in Tregs within the TME also contribute to anti-PD-1 therapy resistance (Kumagai et al, 2020).

Myeloid cells are also affected by the lipid compartment. In liver metastases, M2 macrophages engulf tumor cell-derived long-chain fatty acids via CD36 and enhance tumor-promoting activities (Yang et al, 2022). It has been shown that the accumulation of lipids in dendritic cells (DCs) causes the failure of tumor-associated antigen presentation through endoplasmic reticulum (ER) stress and its response factor, XBP1 (Cubillos-Ruiz et al, 2015; Herber et al, 2010). Moreover, DCs educated by FASN-elevated tumor cells also have defects in T cell priming (Jiang et al, 2018). Glycerol can be generated from the glycolytic metabolite dihydroxyacetone phosphate (DHAP), and it is an essential backbone for lipogenesis. Like DCs, TAMs also elevate CD36 expression for M2 activation through lipolysis (Huang et al, 2014). Except for canonical FA uptake, TAMs can also sense β-glucosylceramide via the Ca2^+^-dependent lectin receptor, also known as Mincle, which contributes to a pro-tumorigenic phenotype and to survival through ER stress responses (Di Conza et al, 2021). Therefore, there has been considerable interest in targeting ACLY with anti-cancer drugs since many cancer cells rely on its activity in fatty acid metabolism, cholesterol biosynthesis, and protein acetylation and prenylation (Hatzivassiliou et al, 2005; Zaidi et al, 2012).

### Immune–tumor interactions contribute to tumor heterogeneity and immune surveillance

The interplay between tumor cells and infiltrated immune cells is persistent, dynamic, and evolving from the initial establishment of scarce cancer cells or clones to the aggressive disease. Studies have shown that the composition of immune cells in distant metastatic sites influences the ability of tumor cells to acquire immune-evasive capacity during metastasis (Baumann et al, 2022). Due to oncogenic drivers or external stimuli, tumor cells are usually capable of plasticity manifested by an EMT phenotype, which has been implicated in resistance mechanisms to ICB therapy (Chen et al, 2014; Hugo et al, 2016; Mak et al, 2016). When tumor cells go through EMT, they are affected by transcription factors such as Snail, Slug, and Zeb1/2, as well as various cytokines, including TGF-β and IL-8 (Guinney et al, 2015). Cancer-derived IL-8 likely recruits myeloid-derived suppressive cells, which exclude and suppress effector T cells (Schalper et al, 2020; Yuen et al, 2020). While TGF-β is produced by tumor cells and by several other cell types, including Tregs, macrophages, and fibroblasts, elevated TGF-β levels favor naive T cell differentiation towards the Treg subset and dampen antigen-presenting abilities of dendritic cells (Flavell et al, 2010). Further studies have shown that the expression of Zeb1 triggers immune checkpoints, such as PDL1 and CD47 checkpoints, to establish an immunosuppressive TME (Guo et al, 2021). Additionally, the expression of EMT transcription factors in cancer cells can dampen killing mediated by T and NK cells (Akalay et al, 2013; David et al, 2016; Hamilton et al, 2014; Kudo-Saito et al, 2009; Terry et al, 2017).

The heterogeneity of the TME goes beyond the genetic differences within tumor cells and encourages tumor cells to spread and hinder the host immune responses. For example, neutrophil infiltration increases Snail expression, inhibits immunotherapy efficacy, and alters angiogenesis, which enhances an amplification loop favoring metastasis through neutrophil recruitment (Faget et al, 2017). Moreover, tumor-associated neutrophils can produce IL-17a to promote cancer EMT through JAK2/STAT3 signaling (Li et al, 2019b). Overall, the current understandings propose that there is a connection between high immune suppression and the development of tumor plasticity, which causes cancer heterogeneity and leads to a poorer prognosis for various types of cancer.

In many different types of cancers, the presence of tumor-infiltrating T cells within the tumor can predict patient survival (Clemente et al, 1996; Epstein and Fatti, 1976; Jass, 1986; Lipponen et al, 1992; Naito et al, 1998; Rilke et al, 1991; Schumacher et al, 2001; Zhang et al, 2003). This suggests that the immune system can defend against infections and abnormal cells, indicating the possibility of cancer immunosurveillance. However, this raises the question of how cancer cells manage to avoid detection. Approximately two decades ago, Robert Schreibers team discovered that IFNγ and lymphocytes not only protect the host against tumor growth by upregulating MHC class I but also function to select for tumor variants with lower immunogenicity, which can more easily evade immunosurveillance (Shankaran et al, 2001). Accordingly, they proposed an immunoediting process whereby tumor antigenicity can be imprinted by the immunologic environment in which they develop (Dunn et al, 2002). The theory implies that the immune system protects host from the development of malignancy, but it also contributes to sculpturing immunogenicity of tumors and acquiring the capacity to evade immunosurveillance.

This dynamic process of immunoediting is proposed to consist of three stages: elimination, equilibrium, and escape, whereby the immune system can constrain and promote tumor development. In the elimination stage, abnormal cells are sensed by innate and adaptive immunity, particularly in NKs and CD8^+^ T cells, which control the development of tumors. Additionally, the binding of stress-induced ligands on the surface of cancer cells to the NKG2D receptor of NK and cytotoxic T cells prompt the secretion of immunomodulatory and pro-inflammatory cytokines, such as IFNγ, which further stimulate immunosurveillance (Guerra et al, 2008). The second stage corresponds to a balance of fight-tug between tumor and immune cells. Hence, the immune system cannot eliminate the tumor cells due to generated cancer cell clones with reduced immunogenicity, usually because of genomic instability. However, the tumor growth is in check by immune system, and the tumor exhibits a property of dormancy due to the balance of tumor-suppressing and tumor-promoting factors. CTCs can rewire their metabolism to sustain the arrest demands and management of the new stationary state at metastatic sites. A previous study has shown that cancer cells that are capable of latency can become dormant in primary and metastatic organs for long periods by avoiding detection from the innate immune system, especially NK cell-mediated clearance (Malladi et al, 2016). Correspondingly, mice with dormant sarcomas had significantly higher rates of NK cells than those with progressing sarcomas (Wu et al, 2013). A previous study has shown that lymphocyte-derived IFNγ and TNF within tumors can induce senescence in numerous murine and human cancers, which may be one of the mechanisms for the dormancy (Braumuller et al, 2013). As addressed previously, IFNγ mainly secreted by CD8^**+**^ T cells and NK cells, could induce dormancy and G0/G1 growth arrest in cancer cell via STAT1 signaling (Aqbi et al, 2018; Dimco et al, 2010; Kortylewski et al, 2004). In B-cell lymphoma, CD8^**+**^ T cell deprivation significantly decreases the period of dormancy and shortens the time for recurrence (Farrar et al, 1999). Apart from CD8^**+**^ T cells, CD4^**+**^ T cells also cooperate with IFNγ signaling through TNFα-p55 axis to induce tumor dormancy in a pancreatic cancer mouse model (Muller-Hermelink et al, 2008).

When the immune system cannot destroy cancer, which turns into the escape stage, tumor develops with a clinically detectable tumor appearance. During this phase, tumor cell variants emerge, which are less immunogenic and contain immune-resistant features. These changes can reduce antigen expression on the surface of tumor cells. With cancer progression, immune evasion can happen due to IFNγ-triggered genetic instability before tumors become more invasive, suggesting immune cell edit tumor antigenicity (Mascaux et al, 2019; Takeda et al, 2017). Besides the loss of antigens, IFNγ facilitates immune escape by driving expression of PD-L1 in tumor cells to hamper anti-tumor immune responses through PD-L1/PD-1 axis-mediated dysfunctional TILs (Dong et al, 2002; Garcia-Diaz et al, 2017). Moreover, IFNγ expression can diminish NKG2D ligands on cancer cells to avoid NK cell-based killing (Bui et al, 2006; O’Sullivan et al, 2011). More recently, immunoediting also contributed to the metastatic phenotype, indicating that NK cells guide the fate of the circulating tumor cells for EMT and impact metastatic clonal evolution by favoring polyclonal seeding (Lo et al, 2020). In contrast to synchronous brain metastases of breast cancer, latent or metachronous metastatic cancer cells survive in equilibrium with innate immunosurveillance and maintain cellular redox homeostasis through the upregulation of amino acid transporters (Lim et al, 2019; Parida et al, 2022).

Understanding the specific actions of immunoediting is crucial for effective cancer treatment. In fact, the presence of a particular subset of IFN-stimulated genes (ISGs) in cancer cells is associated with immune suppression and resistance to ICB by regulating chromatin accessibility, while abrogating Type I IFN or IFNγ signaling in tumor cells improves the function of many immune compartments, such as CD8^**+**^ T cells, NK cells, and innate lymphoid cells (Benci et al, 2019; Benci et al, 2016; Qiu et al, 2023; Teijaro et al, 2013; Wilson et al, 2013). Accordingly, immunoediting is an ongoing and changing process throughout tumor development that significantly affects the effectiveness of immunotherapy and the chances of cancer relapse.

### Metabolic reprogramming and immune evasion

To survive in a nutrient-deficient or competitive TME, cancer cells need to adapt their metabolism, whereas the immune system in tumors can transmit signals to cells in the vicinity of cancer as well as in distant organs to assist their metabolic reprogramming. Metabolic regulators, for example, AMPK, AKT/mTOR, and MYC, are controlled by various factors within the microenvironment, such as cytokines, glucose, amino acid, and growth factors (Dejure and Eilers, 2017; El-Sahli and Wang, 2020; Ivashkiv, 2018; Weichhart et al, 2015). The activation of mTORC2 can promote the expression of AKT and further support glutamine metabolism, ultimately causing chemotherapy resistance and promoting cancer growth and development (Moloughney et al, 2016; Tanaka et al, 2011). In addition, activation of AKT-mTOR pathway liberates PD-L1 expression and regulates T cell infiltration (Lastwika et al, 2016). On the other hand, the balance of energy production, particularly in AMP/ATP ratio, determines the activation of AMPK, and the AMPK axis can increase the uptake of fatty acids and glucose as well as their catabolism capacity to elevate the intracellular ATP level (Samovski et al, 2015). Certain immune cell-derived cytokines, like IL-1, IL-17, IL-18, and IL-22, can impact metabolic regulators and contribute to immunosuppression (Briukhovetska et al, 2021). Tumor metabolism is not constant but changes as the tumor evolves and responds to shifts in the TME. Hence, tumor cells alter their metabolism in certain situations to exploit resources and evade immune response.

In addition to immunogenicity reduction, emerging evidence highlights that tumor cells may impose metabolic stress in the TME by depleting nutrients and facilitating accumulation of by-product metabolites known to modulate functions of immune cells (Ho and Liu, 2016; Li et al, 2019c). Hence, the insights into the mechanisms of tumor metabolic adaption during immunosurveillance point towards bidirectional crosstalk between immune cells and cancer cells as a crucial regulatory factor (Kao et al, 2022). Metabolic adaptation of tumor cells by presenting a more glycolytic metabolism leads to a stress microenvironment, such as lactate enrichment, hypoxia development, and nutrient deficiency (Villalba et al, 2013). These changes can induce expression of stress markers, e.g., UL16 binding proteins (ULBPs) or MHC class I polypeptide-related sequence A/B (MICA/B), which are recognized by cytotoxic lymphocytes (Liu et al, 2019). MICA expression in the plasma membrane is coupled to lactate, short-chain fatty acids, glycolysis, and purine nucleotide synthesis (McCarthy et al, 2018; Andresen et al, 2009). In detail, exposure to short-chain fatty acids promotes histone acetylation and induces MICA/B expression by increasing the availability of acetyl-CoA, which links the metabolic state to epigenetics for gene regulation (Hogh et al, 2020). Together, metabolic stress can alter the intrinsic cellular signaling or reprogram epigenomics, affecting the functionality of surrounding immune cells.

Notably, heterogenous tumors cause metabolic complications and immune evasion through competition. For example, increased glycolysis in tumor cells can inhibit cytotoxic lymphocyte function by downregulating MHC class I expression (Catalan et al, 2015; Siska et al, 2020). The infiltration of immune cells appears to control metabolic process of nearby cancer cells, which facilitates cancer progression and immune resistance to adoptive cellular therapy (Cascone et al, 2018). IFNγ expressed by CD8 T cells may induce the expression of immunosuppressive metabolic enzymes such as indoleamine-2, 3-deoxygenase (IDO), a tryptophan-metabolizing enzyme that negatively affects effector T cell function (Gajewski et al, 2013). IFNγ expression can also lead to tumor-repopulating cells entering dormancy through upregulation of IDO1/aryl hydrocarbon receptor (AhR) dependent-p27 induction but prevents apoptosis (Liu et al, 2017). Additionally, it has been shown that tumor-associated macrophages can contribute to the growth of encountered cancer cells by promoting their glycolysis through TNFα (Jeong et al, 2019). Apart from this, CD39 and CD73 enzymes, which are the downstream targets of STAT3 signaling, coordinately dephosphorylate adenosine triphosphate (ATP) to form adenosine, thus promoting tumor cell metastasis (Mittal et al, 2016; Stagg et al, 2010) and also angiogenesis (Allard et al, 2014). Adenosine was shown to suppress T cell proliferation and cytotoxic function by activating the A2A receptor (Zhang et al, 2004). CD73 has the potential to be a biomarker for anti-PD-1 therapy. Its high expression can limit the efficacy of anti-PD-1 therapy, but this can be improved by using A2A blockade simultaneously (Beavis et al, 2015). In various types of cancer, high CD73 expression is linked to poor prognosis. Thus, CD73 blockade is being developed as a treatment for cancer (Leclerc et al, 2016; Loi et al, 2013; Turcotte et al, 2015).

### Metabolism-guided immunoediting

All shreds of evidence directed to the idea that the immune system could shape the immunogenic phenotype of tumors, although a unifying mechanism was elusive. The dynamic competition between heterogeneity of tumor cells and immunity against malignant cells determines the spatial and temporal prevalence of tumor cell subclones throughout metastatic disease (Liu et al, 2021). CD8^**+**^ T cells can exert selective pressures on tumor cells, which is the driving force for clonal evolution (Koebel et al, 2007; Liu et al, 2021; McGranahan et al, 2017). Moreover, CD8^**+**^ T cells in the metastatic region also determine whether disseminated tumor cells are eliminated or become dormant (Tallon de Lara et al, 2022; Vitale et al, 2021).

**Figure 3:**
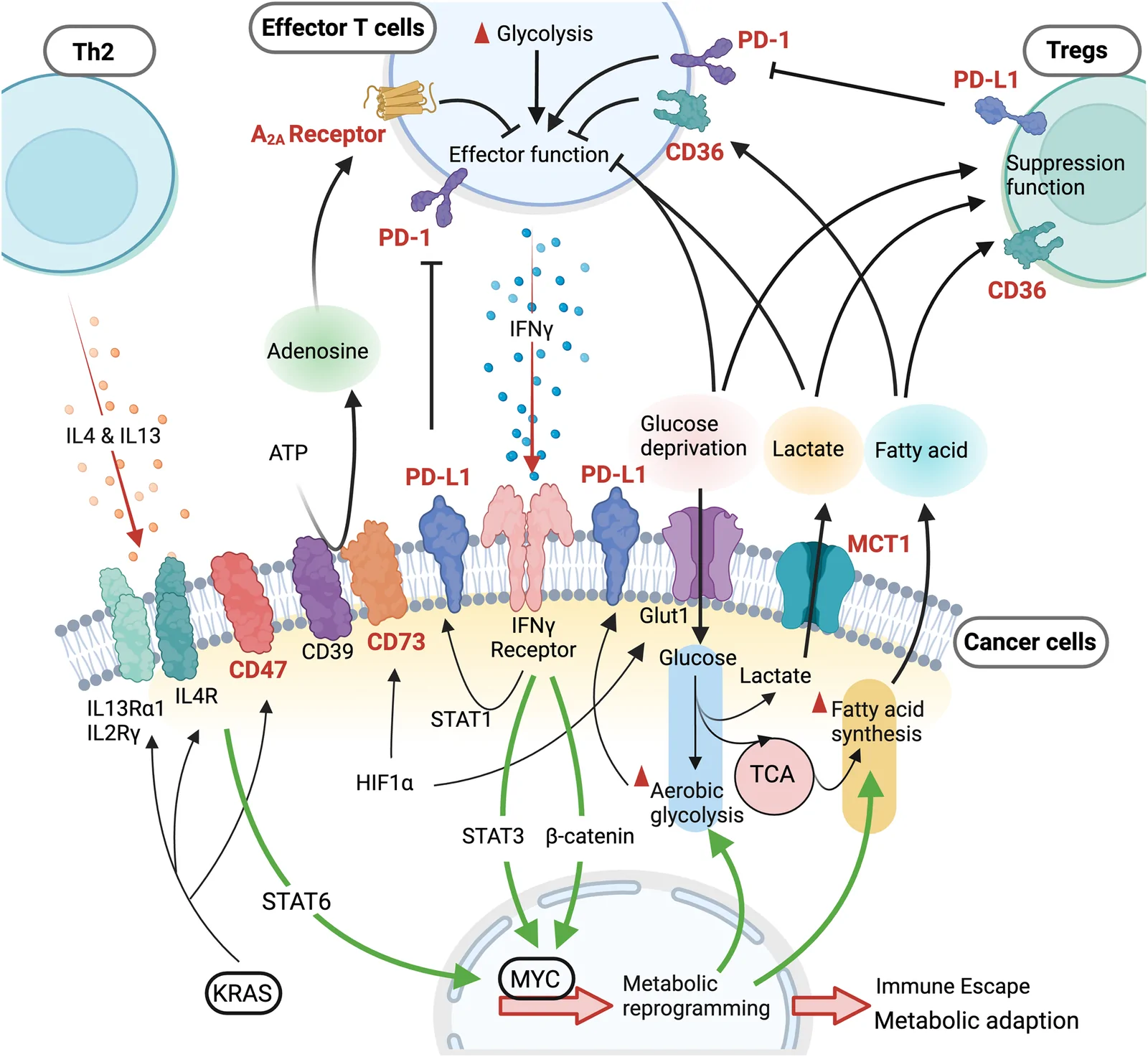
Immune cell-guided metabolic reprogramming leads to immune evasion. During immunosurveillance, a two-way communication occurs between tumor cells and infiltrating immune cells through cytokines or metabolites, controlling immune evasion. Multiple metabolic features contribute to an immune suppressive TME, such as adenosine expression, lactate generation from robust glycolysis, competition for glucose, and fatty acid production. IL4 and IL13 production in Th2 and IFNγ expression from CD8^**+**^ T cells sculpt the cancer cell immunogenicity by reprograming metabolism via epigenetic control (Dey et al, 2020; Li et al, 2023; Tsai et al, 2023). Hence, a metabolic immunoediting process facilitates cancer progression and contributes to escape from immune surveillance by suppressing T cell effector function and promoting survival and function of regulatory T cells (Tregs). The highlighted targets in red and bold represent potential metabolic targets that could be used for clinical cancer therapy. Green lines represent potential pathways for metabolic reprogramming during immune evasion.

Among soluble factors from TILs, cytokines and metabolites within a tumor are the most potential mediators in regulating immune editing. It is increasingly recognized that epigenetics regulation bridges cellular metabolism with immune surveillance in the TME. Cell-permeable α-KG can alter the DNA methylation profile to polarizing Treg conditions (Matias et al, 2021). Additionally, the previous study has shown that palmitic acid, a metabolite, can stimulate H3K4 methylation in CD36-expressing cells, allowing transcriptomic changes (Pascual et al, 2021). MYC, a metabolic and epigenetic regulator, can also orchestrate immune suppressive stromal changes, including innate and adaptive immunity, mainly by presenting CCL9 and IL-23 (Kortlever et al, 2017). The presence of IFNγ can trigger epigenetic reprogramming in human melanoma cells (Kim et al, 2021) and rapidly and transiently increase MYC expression (Ramana et al, 2000). Furthermore, type I interferons can function as resistance hubs by promoting the epigenetic regulator demethylase 1B (KDM1B) to rewire cancer cells for stemness and immune evasion during immunogenic chemotherapy (Musella et al, 2022). Thus, metabolic regulation may induce dormancy in tumorigenic cells and affect anti-tumor immunity, highlighting another potential mechanism of metabolism-driven immune editing through epigenetic modification. To support this, our recent study has shown that IFNγ secreted by TILs within tumors impairs T cell function to induce immune escape by reprograming metabolism via STAT3/MYC signaling, featuring increased aerobic glycolysis and fatty acid synthesis (Tsai et al, 2023). Consistent with this, Li et al also found that TILs-derived IFNγ causes oncometabolic reprogramming in cancer cells, including an increase in aerobic glycolysis and MYC expression via FGF2 signaling. As a result, PKM2 activity is suppressed, and NAD^+^ is decreased, leading to enhanced β-catenin acetylation and faster cancer progression (Li et al, 2023). Additionally, invading Th2 cells in PDAC with KRAS mutation express IL4 or IL13 cytokines and activate type I cytokine receptor complexes signal (IL2rγ–IL4rα and IL2rγ–IL13rα1) to drive MYC mediated glycolysis via STAT6 (Dey et al, 2020). Another study also indicated that the presence of IFNγ from T cell or NK cells can reprogram cellular lipidomes and fatty acid metabolism, which further affects the sensitivity to ferroptosis by regulating the acyl-CoA synthetase long-chain family member 4 (Liao et al, 2022). Thus, as cancer progresses, metabolic adaption allows tumor cells to sustain cell survival in fluctuating nutrient environments, which may also impact immune surveillance. Together, this suggests that cytokines released by immune cells might control the metabolic cascade for immune escape in cancer, suggesting the contribution of metabolic reprogramming in immunoediting and cancer treatment (Fig. 3).

## Targeting metabolism for rewiring anti-tumor immunity

The metabolic tug-of-war between TILs and malignant cells highly impacts the immune surveillance and immunotherapy response (Ho and Liu, 2016; Li et al, 2019c). Several clinical trials are now underway, with a focus on metabolic activity as a target to treat cancer, showing favorable responses to cancer immunotherapy. Strategies such as CD73 blockade, arginase vaccination, and the use of mutant IDH inhibitor have shown promising results with good patient tolerance, and are being considered for further phase II/III studies (see Table EV1). Understanding the impact of tumor heterogeneity on the associations between immune cells and tumor cells is of significant clinical importance, as immunity can halt cancer progression, either through the natural immune system or immunotherapy. ICB therapy may be particularly effective when rewiring the metabolic phenotype of effector T cells or targeting cancer metabolism to render tumor cells low-glycolytic activity or low LDHA-expression, thus enabling activated CD8^**+**^ T cells to manifest within the tumor microenvironment (Patsoukis et al, 2015). It has been reported that ICB has an inhibitory effect on immune cell metabolism by suppressing glycolysis and increasing FAO and lipolysis (Qorraj et al, 2017). This suggests that combining ICB with metabolic interventions could be an alternative approach to improve the antitumor effects by reversing immune metabolic dysfunctions. For hepatocellular carcinomas with a deficient urea cycle, the combined treatment of arginine restriction and GCN2 inhibition significantly exhibits tumor supersession (Missiaen et al, 2022). Treatment of dichloroacetate (DCA), which can promote oxidative phosphorylation in p53 positive tumor cells, increases the effectiveness of CAR T-cell or allogeneic NK cell therapies by inducing the expression of stress ligands, such as MICA/B (Belkahla et al, 2022). In a B16 melanoma mouse model, metformin promoted efficacy of PD-1 blockade by reinvigorating T cells (Scharping et al, 2017). These findings highlight the potential of targeting cancer metabolism to boost current immune therapy and avoid recurrence. Therefore, treating highly resistant tumor cells in refractory patients requires using metabolic drugs or nutrient restrictions that sensitize tumor cells to cytotoxic lymphocytes (Brenner et al, 2020). However, there is still room for improvement in the bench-to-bedside process for metabolic targeting therapy. For instance, the application of an IDO blocker to the current ICB did not result in improved efficacy (Table EV1).

As they progress, tumor cells become more diverse due to genomic instability and acquired resistance to treatments, such as target therapy or ICB. For example, lung tumors overcome targeting therapy of EGFR inhibitor by presenting downstream mutation or alternative signaling cascade to confer resistance (Hata et al, 2016; Russo et al, 2016). Therefore, a deeper understanding of the relationship between signaling cascades and metabolic networks may provide the potential to develop novel combinatorial therapeutic strategies in this context. Remarkably, tumors displaying metabolic plasticity might be targeted by combining kinase inhibitors and the metabolic drug, which exhibits a synergistic effect (Hulea et al, 2018). Furthermore, context-dependent immune profiles and metabolic features within the heterogeneous tumor or between individual patients could affect bench-to-bedside clinical success. For instance, APC- or MYC-driven pancreatic tumors were sensitive to depletion of serine and glycine, leading to reduced growth; in contrast, KRAS-driven tumors were not affected by serine and glycine removal (Maddocks et al, 2017).

Targeting cancer metabolism still presents challenges as some metabolic interventions may further suppress immune surveillance, which relies on those metabolic processes. When T cells are activated, they switch to aerobic glycolysis, increasing glucose and glutamine uptake, which boosts their rapid expansion and cytotoxic activity (Pacella et al, 2018). However, inhibiting glycolysis in cancer can also render T cells quiescent status. Additionally, due to the differential requirement for glutamine, completely blocking glutamine uptake in tumors can lead to Tregs development, limiting T cell-based therapy. The mechanism behind how tumors speed up their growth during ICB and immunosurveillance remains unclear. Cancer patients can display disease evolution, such as hyperprogressive disease, upon ICB immunotherapy, suggesting ICB or metabolic therapy could trigger a chain reaction that alters the immune response, including immune cell differentiation and cytokine profiles, leading to another round of immunoediting and ultimately treatment resistance. Consequently, the outcome of cancer therapy and the required strategy can be influenced by immune therapies like CAR T-cells and ICB, as well as the presence of bystander T cells. These parameters can shift the immune cell landscape and increase selection pressure, resulting in further clone evolution in cancer. In addition, pre-conditioning metabolic state within the TME and in TILs has been suggested to be a critical requirement for effective anti-tumor responses induced by treatments such as agonistic anti-CD40 antibodies (Liu et al, 2023). It is worth noting that immune exclusion, which causes resistance to ICB, can result in a different metabolic tumor microenvironment due to reduced levels of infiltrating CD8^**+**^ T cells and IFNγ gene signature, which also impact the outcome of metabolic innervation. Thus, it is crucial to identify the unique metabolic nodes that tumor cells use to evade the immune system (see also Box 1). By doing so, we can determine the optimal time to alter immunometabolism and cancer metabolism, which could significantly improve current immunotherapy methods.

In need of answers

- How do tumor-derived metabolites or tumor metabolism impact adjacent stromal cells and systematic immune profiles?
- How do metabolites guide cell differentiation or immune escape in a cell type-specific manner?
- What are the translational limitations of metabolism-targeting therapies moving from animal models to the human context?
- What is the impact of diverse immune cells on immune escape and immune therapy during different stages of cancer progression?

## Conclusions

Robust data show that metabolic reprogramming is particularly beneficial when tumor cells encounter selective pressures, such as metastatic barriers, fluctuating nutrient environments, and immune surveillance. Although the metabolic plasticity of cancer cells may promote metastatic colonization, seeding at specific secondary sites may require CTCs to meet metabolic and nutritional demands in TME. As cancer spreads from the primary tumor to distant sites, the cells that are selected to colonize specific tissues and evade the immune system may be determined by their metabolic programs. This means that different populations of cells within the primary tumor may have distinct metabolic features that help them to survive and to grow in different environments. On the other hand, environmental factors, such as hypoxia, unique metabolites, and immunosurveillance, may reprogram the metabolic activity, which triggers cancer cells to enter a dormant state or to experience outgrowth. Investigating microenvironmental influences, immunosurveillance, and metabolic heterogeneity within tumors may lead to further understanding of how metastatic cancers can remain dormant for an extended period before relapse, and this mechanism could be utilized to prevent metastasis (see also Box 1). However, the success of targeting cancer metabolism to reprogram immune states in tumors, restricting tumor growth, and ameliorating tumor metastasis, largely relies on our understanding of the metabolic crosstalk during tumor progression and evolution. The presence of immune cells or of immunotherapeutic interventions seems to prove a selection pressure to edit cancer immunogenicity, tumorigenicity, and metastatic ability through metabolic reprogramming (Liao et al, 2022; Tsai et al, 2023). Thus, further research on this emerging topic would provide a critical steppingstone for effectively harnessing metabolic targeting to cancer treatment.

## Supplementary information

Table EV1
